# Comparative cytogenetics and derived phylogenic relationship among *Sitophilus* grain weevils (Coleoptera, Curculionidae, Dryophthorinae)

**DOI:** 10.3897/CompCytogen.v12i2.26412

**Published:** 2018-07-07

**Authors:** Alexandra Avelar Silva, Lucas Soares Braga, Alberto Soares Corrêa, Valerie Renee Holmes, John Spencer Johnston, Brenda Oppert, Raul Narciso Carvalho Guedes, Mara Garcia Tavares

**Affiliations:** 1 Departamento de Biologia Geral, Universidade Federal de Viçosa, Viçosa, MG 36570-900, Brazil; 2 Departamento de Entomologia, Universidade Federal de Viçosa, Viçosa, MG 36570-900, Brazil; 3 Departamento de Entomologia e Acarologia, Escola Superior de Agricultura “Luiz de Queiroz", Universidade de São Paulo, Piracicaba, SP 13418-900, Brazil; 4 Department of Entomology, Texas A&M University, College Station, TX 77843, USA; 5 USDA-ARS, Center for Grain and Animal Health Research, Manhattan, KS 66506, USA

**Keywords:** karyotypes, C-banding, fluorochromes, heterochromatin, stored products, evolutionary history

## Abstract

Cytogenetic characteristics and genome size are powerful tools for species characterization and identification of cryptic species, providing critical insights into phylogenetic and evolutionary relationships. *Sitophilus* Linnaeus, 1758 grain weevils can benefit from such tools as key pest species of stored products and also as sources of archeological information on human history and past urban environments. Moreover, the phylogenetic relationship among these weevil species remains controversial and is largely based on single DNA fragment analyses. Therefore, cytogenetic analyses and genome size determinations were performed for four *Sitophilus* grain weevil species, namely the granary weevil *Sitophilus granarius* (Linnaeus, 1758), the tamarind weevil *S. linearis* (Herbst, 1797), the rice weevil *S. oryzae* (Linnaeus, 1763), and the maize weevil *S. zeamais* Motschulsky, 1855. Both maize and rice weevils exhibited the same chromosome number (2n=22; 10 A + Xyp). In contrast, the granary and tamarind weevils exhibited higher chromosome number (2n=24; 11 A + Xyp and 11 A + neo-XY, respectively). The nuclear DNA content of these species was not proportionally related to either chromosome number or heterochromatin amount. Maize and rice weevils exhibited similar and larger genome sizes (0.730±0.003 pg and 0.786±0.003 pg, respectively), followed by the granary weevil (0.553±0.003 pg), and the tamarind weevil (0.440±0.001 pg). Parsimony phylogenetic analysis of the insect karyotypes indicate that *S. zeamais* and *S. oryzae* were phylogenetically closer than *S. granarius* and *S. linearis*, which were more closely related and share a more recent ancestral relationship.

## Introduction

Closely related species usually exhibit similar karyotypes concerning chromosome number and morphology. However, other characteristics such as the amount, size and distribution of heterochromatic blocks and/or nucleolus organizing regions (NORs) can vary considerably, even among cryptic species, which makes cytogenetic analyses powerful tools for species characterization and identification (Holecová et al. 2002, Rozek et al. 2004, Lachowska et al. 2004, 2006, 2008, 2009, Angus et al. 2011). As a consequence, these analyses can lead to important insights into phylogenetic relationships and evolutionary history, contributing to the understanding of species context and relevance. Although seldom used, such knowledge is particularly appealing for economically important insect pest species, and/or species that shed light on human history/past urban environments, and grain trade and trade routes, as exemplified by stored product insect pest species (Levinson and Levinson 1994, Kenway and Carrott 2006, Smith and Kenward 2011, Corrêa et al. 2017).

Interspecific divergence is also associated with chromosome variation (Goodisman et al. 2008), encouraging the use of cytogenetic analysis for inferences about the process of chromosome evolution (Sumner 2003). In this context, base-specific fluorochromes and fluorescent *in situ* hybridization (FISH) with different ribosomal DNA probes allow a more detailed analysis of the molecular structure of chromosomes, and reveal many more differences among closely related species than conventional techniques (Bione et al. 2005, Silva et al. 2009, Cabral-de-Mello et al. 2010, 2011). As an example, the identification of rRNA clusters in different species has been widely used in comparative cytogenetics to understand the patterns of karyotypic evolution in different taxonomic groups (Cuadrado et al. 2008, Cabral-de-Mello et al. 2011, Cioffi et al. 2011, Grozeva et al. 2011, Golub et al. 2015, Palacios-Gimenez and Cabral-de-Mello 2015).

Genome size is another trait useful in comparative studies in a variety of taxonomic levels (Gregory and Shorthouse 2003, Tsutsui et al. 2008, Tavares et al. 2012). Such information is also important to clarify the relationship between variation in genome size and chromosome number (Tsutsui et al. 2008, Cardoso et al. 2012, Jacobson et al. 2012), and direct the selection of species for genome sequencing projects (Hardie et al. 2002, Gregory 2005, Geraci et al. 2007).

Curiously, cytogenetic studies are non-existent for several taxa and species groups that have recognized importance as pest species, and exhibit archaeological relevance, such as grain weevils of the genus *Sitophilus* Linnaeus, 1758 (Kenway and Carrott 2006, Plarre 2010, Smith and Kenward 2011, Corrêa et al. 2017). A few species of *Sitophilus* weevils were karyotyped to date, mainly in the 1970’s and 1980’s (Inkmann 1933, cited in Smith and Virkki 1978, Takenouchi 1958, cited in Smith and Virkki 1978, Smith and Brower 1974, Smith and Virkki 1978, Barrion et al. 1988, Zhi-Yua et al. 1989, Moraes et al. 2003, Silva et al. 2015). However, the results of these earlier efforts involving grain weevils were discrepant, emphasizing the need for further and more reliable analysis. Only a single recent karyotype analysis of the maize weevil *Sitophilus zeamais* Motschulsky, 1855 used more refined cytogenetic techniques (Silva et al. 2015). Knowledge of genome size is even scarcer, since no data are currently available in the literature for any species of *Sitophilus*.

The genus *Sitophilus* comprises fourteen species, three of which (the rice weevil *S. oryzae* (Linnaeus, 1763), the maize weevil *S. zeamais* and the granary weevil *S. granarius* (Linnaeus, 1758)), are of greater scientific interest because of their broadly recognized status as primary pest species of stored products throughout the world (Rees 1996, Danho et al. 2002, Ojo and Omoloye 2012). However, a congeneric fourth species, the tamarind weevil *S. linearis* (Herbst 1797), is also of scientific interest due to its devastating seed damage to tamarind crops (*Tamarindus indica* L.) (Adebayo et al. 2011, Ojo and Omoloye 2015).

The phylogenetic relationship among these weevils is controversial (Khan and Musgrave 1968, Plarre 2010). Sequencing-based molecular analyses of individual gene fragments, particularly those encoding cytocrome oxidase I, the elongation factor 1-alpha, and ribosome 28S provided the basis for the initial suggestion that *S. granarius* and *S. zeamais* form a sister taxon to *S. oryzae*, with *S. linearis* more distantly related (O’Meara 2001, Plarre 2010). Alternatively, the granary weevil was reported as a sister species of *S. oryzae*/*S. zeamais* (Lefevre et al. 2004), while in another study, *S. oryzae* and *S. granarius* form the sister group of *S. zeamais* (Conord et al. 2008). *Sitophilus linearis* was also considered a sister group of *S.oryzae/S. zeamais*, not *S. granarius*, in a recent study (Devi et al. 2017). Considering these difficulties and the resulting controversy, cytogenetic analyses and genome size determinations are needed to shed light on the phylogenetic relationship among these *Sitophilus* species.

The aims of this study were to: 1) perform a comparative cytogenetic characterization among *S. granarius*, *S. linearis*, *S. oryzae* and *S. zeamais*); 2) quantify the genome size of these four species; and 3) perform a more complete karyotype-based phylogenetic analysis with these species. The data will contribute to the understanding of the genomic organization and the taxonomic status of these species.

## Materials and methods

### Biological material

*Sitophilus granarius* were obtained from wheat kernels in Manhattan (Kansas, USA; 39°11'18"N; 96°36'21"W); *S. linearis* was obtained from tamarind seeds in Piracicaba (São Paulo, Brazil; 22°43'31"S; 47°38'57"W) and Montes Claros (Minas Gerais, Brazil; 16°44'06"S; 43°51'42"W); and *S. oryzae* was obtained from rice kernels in Cascavel (Paraná, Brazil; 24°57'21"S; 53°27'19"W) and São Borja (Rio Grande do Sul; Brazil; 28°39'38"S; 56°00'16"W). Samples of *S. zeamais* were obtained from maize kernels in Cruzeiro do Sul (Acre, Brazil; 07°37'52"S; 72°40'12"W) and Porto Alegre (Rio Grande do Sul, Brazil; 30°01'59"S; 51°13'48"W).

The last larval instars of each weevil species (i.e., *Sitophilus granarius*, *S. linearis*, *S. oryzae* and *S. zeamais*) were used for karyotyping and adult insects were used for genome size determination. Insects of each species were reared in glass containers (0.5 L) in an environmentally controlled rearing room (18 ± 2 °C, 70 ± 10% relative humidity and a photoperiod of 12:12 h L:D), containing grains of either wheat (*S. granarius*), tamarind fruits (*S. linearis*) or maize grains (*S. oryzae* and *S. zeamais*). The larvae were extracted from their respective hosts after inspection of different substrate grains with a LX-60 specimen radiography system equipped with a 14-bit digital camera (Faxitron X-Ray Corp., Wheeling, IL, USA). The adults were sieved from the grains, snap-frozen in dry ice and maintained under –80 °C until genome size determination.

### Cytogenetic analyses

The cerebral ganglia of individuals of the last larval stage were processed according to Imai et al. (1988) after incubation in a hypotonic solution of colchicine (1% sodium citrate plus 0.005% colchicine) for 1 h 45 min. Conventional staining of the slides was performed with 4% Giemsa in Sörensen`s phosphate buffer pH 6.8, for 12 min. Slides were then washed in water and allowed to dry at room temperature. The C-banding technique was performed according to Lachowska et al. (2005), with modifications to the time of the HCl treatment (0.3M, for 4 min) and the Ba(OH)_2_ incubation (5%, for 3 min). Sequential staining with the fluorochrome DAPI/CMA_3_ was performed according to Schweizer (1980), with modifications related to the order of use of fluorochromes and the processing times. DAPI was used first for 30 min, followed by CMA_3_ for 1 h. The use of distamycin was omitted.

Mapping of ribosomal DNA was performed with probes for 18S rDNA obtained by PCR amplification using primers F (5’ TCATATGCTTGTCTAAAGA-3’) and R (3’-TCTAATTTTTTCAAAGTAAACGC-5’) designed for *Melipona quinquefasciata* Lepeletier, 1836 (Pereira 2006). During the amplification, the 18S rDNA probes were labeled by the indirect method using digoxigenin-11-dUTP (Roche, Mannheim, Germany). Fluorescent in situ hybridization (FISH) was performed using the method proposed by Pinkel et al. (1986), with modifications concerning the use of pepsin instead of proteinase K, before the dehydration and denaturation steps. The detection of the probe signal was achieved with antidigoxigenin-rhodamine. At the end, the slides were mounted with antifading mounting media containing DAPI (Vectashield).

The sex chromosomes were identified by comparing female and male karyotypes. Ten male karyotypes of each species were mounted in order to establish which chromosomes do not form an exact pair. These chromosomes were considered the sex ones and, by comparison, it was possible to establish the chromosomes corresponding to the sex pair, in females. The sex determination system of the four species, in turn, was recognized by analysing meiotic figures from the testes following Dias et al. (2012). Males were identified by the rostrum morphology, which is smaller, thicker and more punctured than the female rostrum (Khan and Musgrave 1968).

An average of 20 metaphases per slide were evaluated with an Olympus BX60 microscope coupled to an image capturing system (Image-Pro Plus Version 6.3, Media Cybernetics 2009). The slides stained with fluorochromes (CMA_3_/DAPI) were analyzed with an epifluorescence light microscope using excitation filters WB (λ = 330–385 nm) and WU (λ = 450–480 nm) under oil immersion at 100× magnification. The chromosomes were classified according to Levan et al. (1964), and the karyotypes were mounted by pairing chromosomes in decreasing order of size.

### Flow cytometry analysis

Genome size was estimated by flow cytometry as described in Hare and Johnston (2011), except that the mean fluorescence of the sample and standard were determined using a Beckman Coulter Cytoflex cytometer and the concentration of propidium iodide was 25μg/ml, rather than 50μg/ml. In brief, a single frozen weevil head plus a single frozen head of a *Drosophila virilis* Sturtevant, 1916 standard (1C = 328 Mbp) were placed into 1ml of Galbraith buffer in a 2 ml Kontes tissue grinder and ground with 15 strokes of the “A" pestle at a rate of 3 strokes per 2 seconds. The nuclei released by grinding were filtered through a 40µ nylon filter and stained with 25 ug/ml of propidium iodide for at least 30 minutes in the cold and dark. The relative fluorescence of the 2C nuclei from each of the four *Sitophilus* species and the standard were determined using the flow cytometer indicated above. The 1C amount of DNA was calculated as the ratio of the mean fluorescence of the diploid nuclei of the sample and standard times 328 Mbp.

### Phylogenetic analysis

The relationship among the four species of *Sitophilus* grain weevils was determined using a matrix with a total of 20 karyotype characters, where five characters were parsimony informative (exhibiting at least two characters distinct among operation taxonomic units [OTUs]; i.e., the weevil species studied) (Table 2). A maximum parsimony (MP) was consequently built using the heuristic search option in the TNT software (Goloboff et al. 2008). Node support was estimated by 100,000 bootstrap replicates using absolute frequency and search tree with implicit enumeration. The vine weevil *Otiorhynchus bisulcatus* (Fabricius, 1781) (Coleoptera: Curculionidae) was the outgroup (Holecová et al. 2013). The maximum parsimony tree shows only nodes with bootstrap support > 50. For the phylogenetic analysis of the chromosomal data each structural rearrangement identified was considered a character and scored for variation among four species and the respective outgroup.

## Results

### Cytogenetics

**Figure 1.**
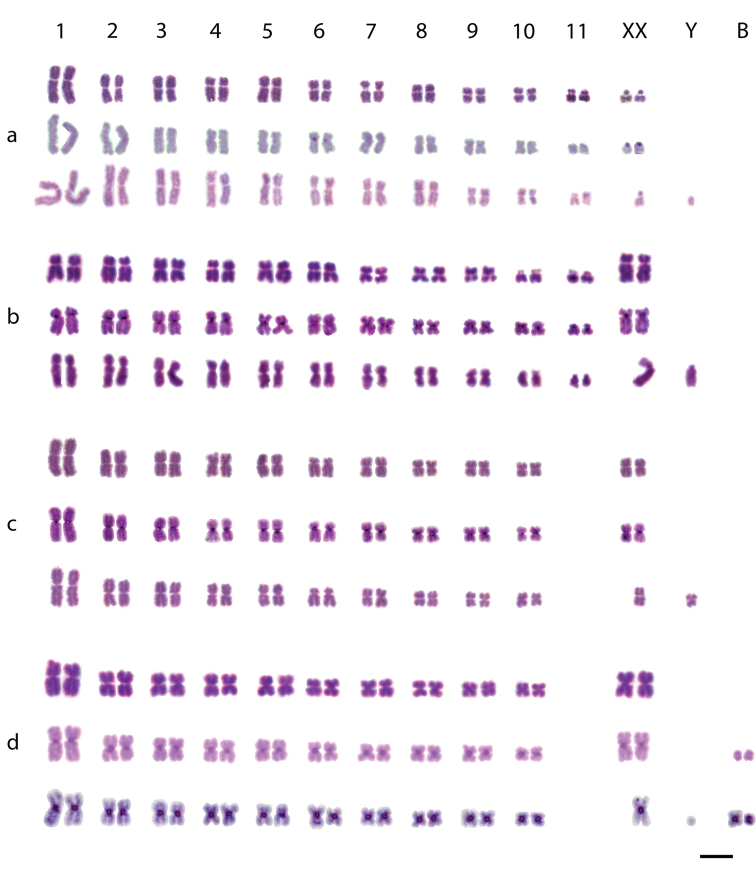
Karyotypes of *Sitophilus granarius* (**a**), *S. linearis* (**b**), *S. oryzae* (**c**) and *S. zeamais* (**d**). The first and the second lines for each species represent female karyotypes stained with Giemsa and C-banding, respectively, while the third line represents male karyotypes stained with Giemsa (**a, b, c**) or C-band (**d**). Bar = 5 μm.

*Sitophilus granarius*:

The karyotype of *S. granarius* showed 2n=24 chromosomes, including 11 pairs of autosomes and a pair of sex chromosomes. Most autosomal pairs, except pairs 1, 4 and 5, exhibited a metacentric morphology. The first autosomal pair was longer than the remaining and the other pairs gradually decrease in size. The submetacentric X chromosome was similar in size to the 11^th^ chromosome pair, while the metacentric Y chromosome was the smallest element in the set (Figures 1a). The heterochromatin, based on the C-banding staining, was restricted to the centromeric region of the 6^th^ autosomal pair (Fig. 1a), to the short arm of the X chromosome and to one of the Y arms.

**Figure 2.**
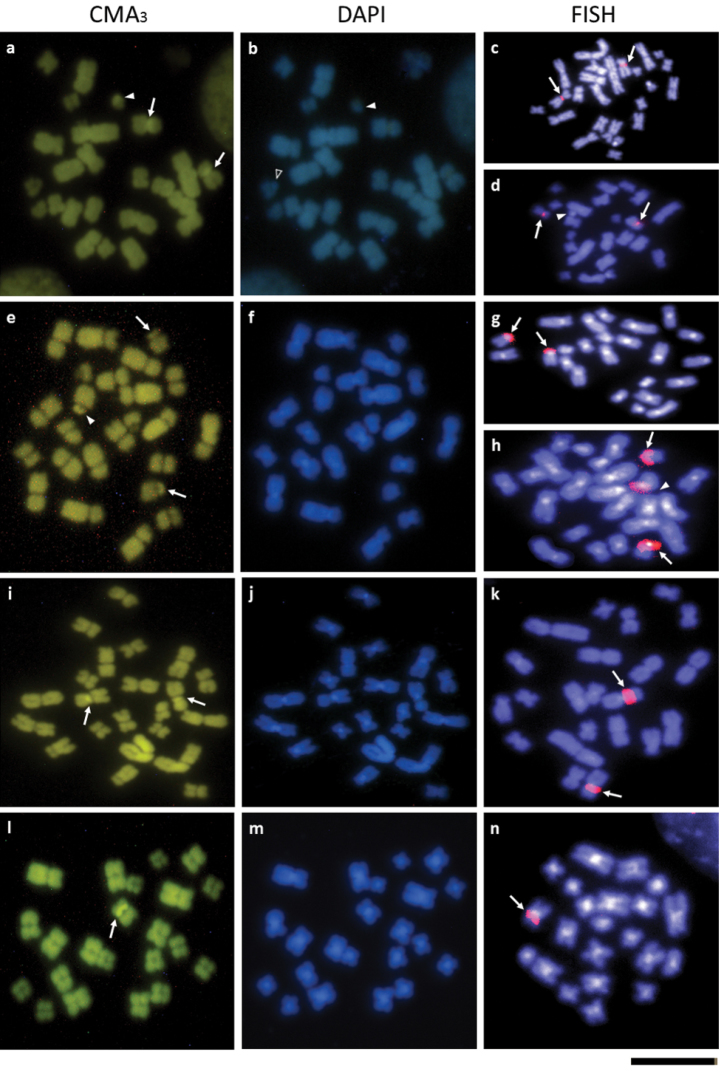
Metaphases of *Sitophilus granarius* (**a–d**), *S. linearis* (**e–h**), *S. oryzae* (**i–k**) and *S. zeamais* (**l–n**) stained with CMA_3_ and DAPI or submitted to rDNA 18S FISH. Pictures **a, b, d, e, f, h** represent male cells, while the remaining ones are from females. The arrows indicate the rDNA location, while blank and solid arrowheads indicate the X and the y chromosomes, respectively. Bar = 5 μm.

Sequential staining with fluorochromes, in turn, allowed the identification of CMA_3_^+^ regions only in the centromere of the sixth autosomal pair and in one of the Y arms, whereas DAPI stained the short arm of the X chromosome and the complementary arm of the Y chromosome (Fig. 2a, b). The FISH technique using an 18S rDNA sequence probe showed a positive hybridization signal in the centromeric region of the sixth autosomal pair, both in males and females (Fig. 2c, d).

**Figure 3.**
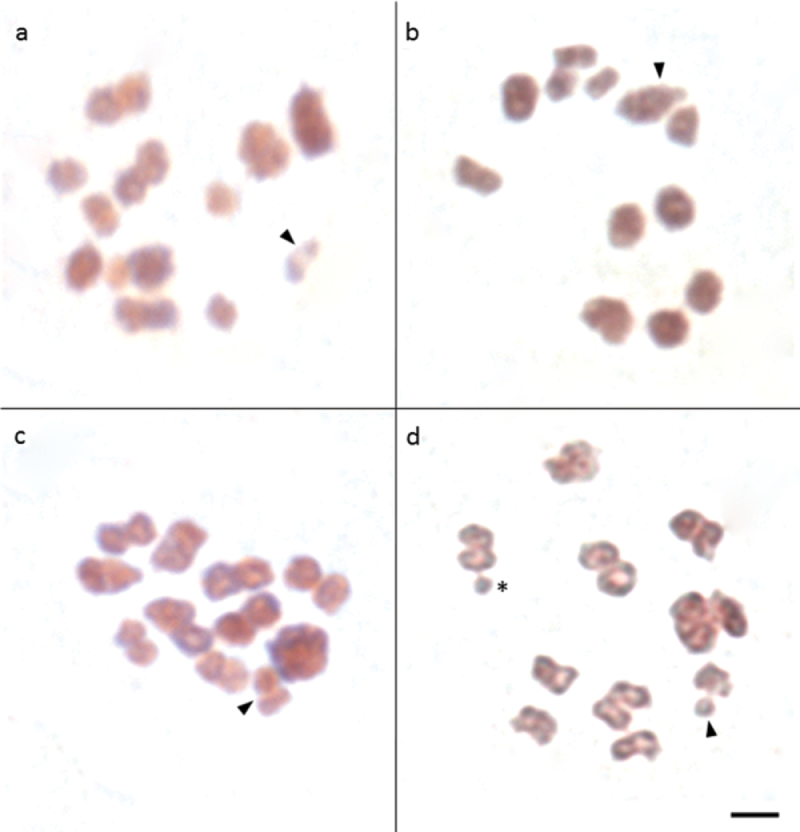
Meiotic male metaphase cells of *Sitophilus granarius* (**a**), *S. linearis* (**b**), *S. oryzae* (**c**) and *S. zeamais* (**d**), stained with Giemsa, showing the typical parachute association of the sex chromosomes (arrowhead) in all species, except in *S. linearis*. The asterisks indicate a B chromosome. Bar = 5 μm.

The analysis of male meiotic cells revealed a sex chromosome system of the Xyp type (Fig. 3a), and the meioformulae n=11 + XX and n=11 + Xyp, observed in females and males respectively.

*Sitophilus linearis*:

The karyotype of this species also exhibited 2n=24 chromosomes, which gradually decrease in size. Most autosomal chromosomes were metacentric, except pairs 1, 2, 10 and 11, which were submetacentric. The submetacentric X chromosome was the longest element in the karyotype, while the Y showed a subtelocentric morphology equal in size to one of the medium-sized chromosomes (Fig. 1b). The C-banding technique showed small heterochromatic blocks in the centromeric region of all chromosomal pairs (Fig. 1b), including the sexual ones, similar to DAPI staining (Fig. 2f). The chromosomal staining with CMA_3_ revealed positive regions located in the telomeric region of pair 10 and in the short arm of the Y chromosome (Fig. 2e).

The chromosomal mapping of major rDNA clusters (18S) confirmed that ribosomal genes were located in the telomeric region of pair 10 and in the short arm of the Y chromosome. So, with both CMA_3_ and FISH, females showed two positive signals, while males showed three positive signals (Fig. 2g, h).

The typical parachute association of the sex chromosomes present in *S. granarius* was not observed, despite the analysis of several metaphase I cells. Instead, analysis of these cells showed an XY association in all cells evaluated (Fig. 3b). Therefore, its meioformulae were n=11 + neo-XX and n=11 + neo-XY, for females and males, respectively.

*Sitophilus oryzae*:

This species exhibited a karyotype consisting of 2n=22 chromosomes that gradually decreased in size. Nine autosomal pairs showed a metacentric morphology; only the autosomal pair 6 was submetacentric (Fig. 1c). The X chromosome was metacentric, presenting an intermediate size between the 7^th^ and 8^th^ chromosome pairs. The Y chromosome was also metacentric, but belonged to the group of the small chromosomes (Fig. 1c). All autosomal chromosomes and the sexual pair possessed small heterochromatic blocks, rich in AT bases in the centromeric region, as showed by the C-banding and the DAPI staining (Figures 1c, 2j). The CMA_3_ staining and the FISH with 18S rDNA indicated that the ribosomal genes were located in the pericentromeric region of the 5^th^ autosomal pair (Figures 2i, k).

Observation of meiotic cells indicated the sex pair exhibiting a parachute configuration, as in *S. granarius*. Therefore, its meioformulae were n=10 + XX and n=10 + Xyp, for females and males, respectively (Fig. 3c).

*Sitophilus zeamais*:

As described by Silva et al. (2015), the karyotype of this species had 2n = 22 chromosomes. All autosomal chromosomes of this species exhibited metacentric morphology and a gradual reduction in size. The X chromosome was also metacentric and presented an intermediate size between the first and second pair of autosomes, while the Y chromosome presented a dot-like morphology (Fig. 1d).

Autosomes and the X chromosome exhibited small heterochromatic blocks in the centromeric region after C-banding and DAPI staining, while the Y chromosome was entirely euchromatic (Figures 1d, 2m). Populations of *S. zeamais* from Viçosa (MG) and Porto Alegre (RS) showed 0-4 B chromosomes that were partially or completely heterochromatic (Fig. 1d). Bright signals were observed in the pericentromeric region of one chromosome of the third autosomal pair after CMA_3_ staining and hybridization with 18S rDNA probe (Figures 2l, n).

Analysis of meiotic cells confirmed that the sex pair exhibited the parachute configuration, as in *S. granarius* and *S. oryzae*. Therefore, their meioformulae were n=10 + XX and 10 + Xyp, for females and males respectively (Fig. 3d).

### Flow cytometry and Phylogenetic Analysis

The mean genome size (1C) estimates for the four *Sitophilus* species analysed in the present study and their chromosome numbers are in Table 1. Genome size was similar between sexes within each species, except when B chromosomes were present in one of the sexes, as in males of the maize weevil *S. zeamais* (Table 1). In contrast, genome size exhibited marked differences among species, which can be clustered in two distinct groups. The 1^st^ group, encompassing *S. granarius* and *S. linearis*, exhibited smaller genome sizes (0.4395–0.5533 pg), while the 2^nd^ group, encompassing *S. oryzae* and *S. zeamais*, exhibited larger genome sizes (0.7296–0.7865 pg). The technique indicated significant variation in genome size of the maize weevil confirming the presence of variable numbers of B chromosomes among specimens of this species and others not possessing them.

**Table 1.** Genome size estimates for the grain weevils *Sitophilus granarius*, *S. linearis*, *S. oryzae* and *S. zeamais*; the number of individuals analyzed (N) and chromosome number are indicated.

Species	Haploid genome size pg ± SE(Mbp ± SE)Female (F) Male (M)		N (F/M)	Chromosome number
*Sitophilus granarius*	0.5533 ± 0.003 (541.1 ± 2.9)	0.5561 ± 0.003 (543.9 ± 3.0)	5/4	2n=24
*Sitophilus linearis*	0.4395 ± 0.001 (429.8 ± 0.6)	0.4351 ± 0.001 (425.5 ± 1.4)	2/4	2n=24
*Sitophilus oryzae*	0.7865 ± 0.002 (769.2 ± 1.9)	0.7852 ± 0.003 (768.0 ± 3.1)	4/6	2n=22
*Sitophilus zeamais*	0.7296 ± 0.008 (713.5 ± 7.5)	0.7252 ± 0.003 (709.2 ± 2.8)0.7860 ± 0.006 (768.7 ± 5.7)	5/3-/2	2n=222n=22 + Bs

**Table 2.** Matrix data of karyotype features of the *Sitophilus* pest species and outgroup *Otiorhynchus bisulcatus* (Coleoptera: Curculionidae).

Karyotype features	Species				
	*S. zeamais*	*S. oryzae*	*S. granarius*	*S. linearis*	O. bisulcatus*
Number of chromosomes	0	0	1	1	0
Presence of B chromosomes	1	0	0	0	0
Sex-chromosome system (Xyp)	1	1	1	0	1
22 metacentric chromosomes	1	0	0	0	0
20 metacentric chromosomes	0	1	0	0	0
18 metacentric chromosomes	0	0	0	1	0
16 metacentric chromosomes	0	0	1	0	1
0 submetacentric chromosomes	1	0	0	0	0
2 submetacentric chromosomes	0	1	0	0	0
8 submetacentric chromosomes	0	0	1	1	0
6 submetacentric chromosomes	0	0	0	1	0
4 submetacentric chromosomes	0	0	0	0	1
1 telocentric chromosome	0	0	0	1	0
Number of the sexual pair	0	1	2	3	?
Morphology of the X chromosome	1	1	0	0	1
Morphology of the y chromosome	0	1	1	2	0
Banda C pattern	0	0	1	0	0
DAPI distribution	0	0	1	0	1
CMA_3_ distribution**	0	1	2	3	4
NOR localization (FISH)**	0	1	2	3	4

*Outgroup obtained of Holecová et al. (2013); **non-informative characters; ?: missing data; 1, 2, 3 and 4: number of variables in chromosome characters.

**Figure 4.**
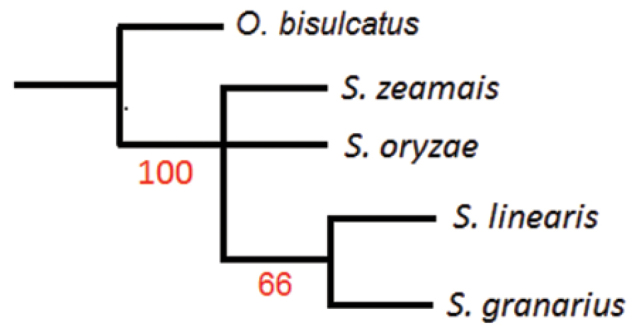
Parsimony tree of *Sitophilus* species with bootstrap values for each node/branch inferred using karyotype traits provided in the Table 2. Node support values below 50% were not recorded in the tree.

The phylogenetic analysis showed that *S. zeamais* and *S. oryzae* were phylogenetically closer than *S. granarius* and *S. linearis*, supported for the clade with bootstrap = 66 (Table 2, Fig. 4). Furthermore, *S. granarius* and *S. linearis* have common and recent ancestry within the genus *Sitophilus*.

## Discussion

### Comparative karyotype characterization

The chromosome number of 2n=22, the parachute configuration, and the prevalence of metacentric chromosomes that we found in *S. oryzae* and *S. zeamais* represent cytogenetic characteristics already described in most species of Curculionidae surveyed so far (Smith and Virkki 1978, Bárcenas-Ortega 1992, Lachowska et al. 1998, 2006, 2008, Holecová et al. 2002, 2013, Rozek et al. 2009). Except for the chromosome number (2n=24), a third species, *S. granarius*, also exhibited karyotypic characteristics likely representing the plesiomorphic (i.e., ancestral) conditions for the Polyphaga suborder of Coleoptera, which are a sex chromosome system of the parachute type (Xyp) and prevalence of metacentric chromosomes (Smith and Virkki 1978, Lachowska et al. 1998, 2006, 2008, Holecová et al. 2002, 2013, Rozek et al. 2009). However, the tamarind weevil, *S. linearis*, exhibited a quite different karyotype from the other three species analysed.

First, the higher number of chromosomes observed in *S. linearis* and *S. granarius* (2n =24) suggests that the karyotype of these species may have evolved by centric fission of autosomes. Alternatively, the karyotypes of *S. oryzae* and *S. zeamais*, that have 2n=22 chromosomes, could have originated as a result of pericentric inversions in small pairs followed by fusions between them. The first scenario, however, seems more probable, once 2n=22 is the prevalent and seems to be the ancestral chromosomal number for Curculionidae species (Smith and Virkki 1978, Holecová et al. 1995, Lachowska et al. 1998). Additionally, centric fission has already been described as playing important roles in the karyotype evolution of other Curculionidae species, such as *Peritelus familiaris* (Lachowska et al. 2006), *Cirrorhynchus kelecsenyi* (Lachowska et al. 2008) and for three sibling species of the *Acalles echinatus* group (i.e., *Acalles echinatus*, *Acalles fallax* and *A. petryszaki*) (Lachowska et al. 2009).

Secondly, cytogenetic analysis revealed differences among the four species related to the morphology and size of sex chromosomes. For example, in *S. granarius* and *S. linearis*, the X chromosome was submetacentric, but the Y chromosome was metacentric and subtelocentric, respectively. In contrast, *S. oryzae* and *S. zeamais* exhibited metacentric X chromosomes, but whereas the Y chromosome in *S. zeamais* was punctiform, that of *S. oryzae* was metacentric and not so small as in *S. zeamais*. In *S. linearis*, in particular, the X chromosome represents the longest element in the karyotype and the Y is also significantly longer than the four/five small autosomes pairs. They are also much larger than the sexual ones in the other three species analysed. Additionally, B chromosomes were found exclusively in some populations of *S. zeamais*. Together, these characteristics facilitate the identification of this particular species.

Thirdly, as the sex chromosomes of *S. linearis* are large and form a well differentiated figure from the Xyp of the other *Sitophilus* species in first meiosis, we propose that this species has a sex determination system of the neo-XY type. However, translocation(s) between an autosomal pair and the sex chromosomes in an ancestral species, with increase of the X-Y sizes and reduction in the number of autosomes, does not seem to explain the origin of the neo-XY system in *S. linearis*. Although the(se) translocation(s) were already observe in some insect species (Macaisne et al 2006, Dutrillaux and Dutrillaux 2007, Mamuris and Dutrillaux 2013), *S. linearis* does not exhibit the reduction in the number of autosomes. Thus, the translocation-based explanation of the origin of the neo-XY system in the tamarind weevil seems flawed. In contrast, this species possesses 2n=24 chromosomes, while the chromosome number of 2n=22 represents the plesiomorphic condition for this genus, as already discussed, what allows for an alternative explanation for the neo-XY system.

A more plausible explanation for the neo-XY system in *S. linearis* would be the contributions of more than one autosomal pair to form the large neo-XY chromosomes, with decreases in their sizes, but without reduction in their number, as reported for *Calcosoma atlas* (Dutrillaux and Dutrillaux 2013). In this sense, cytogenetic analysis provided clear evidence of the absence of the first larger autosome pair in the karyotype *S. linearis*, a characteristic easily recognized in the other three *Sitophilus* species and, consequently, its participation in this process. Additionally, considering the actual size of the sex chromosomes of *S. linearis*, the fact that the two/three first pairs of chromosomes of this species are more similar in size than the equivalent chromosomes in the karyotypes of other *Sitophilus* species, and the diminutive size of the sexual chromosomes of its phylogenetically closer species, *S. granarius* (see below), we can suggest that these chromosomes could also be involved in the formation of the neo-XY chromosomes of *S. linearis*, with small reductions in their sizes. The presence of rDNA clusters in the Y chromosomes of *S. linearis*, as discussed above, is another indication of these translocations. However, further studies will be necessary to confirm this mechanism, the autosomal pairs involved in the process and the exact chromosomal rearrangements concerning the evolution of the neo-sex chromosomes of *S. linearis*.

The genus *Sitophilus*, especially *S. granarius*, possesses a small amount of heterochromatin that was located preferentially at the centromeric region, as in most Curculionidae (Holecová et al. 2002, 2013, Rozek et al. 2004, Lachowska et al. 2005, 2008, 2009, Kajtoch and Lachowska-Cierlik 2009). However, as three of the four species analysed exhibited the same heterochromatic distribution pattern, the C-banding patterns obtained did not allow further discrimination. This finding confirms observations by Rozek et al. (2004) that in species with small amounts of heterochromatin, C-banding patterns cannot be used in taxonomic and phylogenetic investigations. Nonetheless, even considering the consistently and uniquely small heterochromatin amount present in the karyotype of *S. granarius*, the heterochromatin distribution pattern obtained for this species clearly allowed its separation from the other *Sitophilus* species.

The coincidence of DAPI staining with the C-banding marks in the chromosomes of *S. granarius*, *S. linearis* and *S. oryzae*, as well as in *S. zeamais* (Silva et al. 2015), demonstrate the occurrence of a higher amount of AT base pairs in the heterochromatic sequences of these species. Positive DAPI signals were present in the majority of weevils previously studied confirming that AT pairs often make up the main part of the heterochromatin in these species (Lachowska 2008, Lachowska et al. 2008, Holecová et al. 2013). Up to now, Otiorhynchus s. str. bisulcatus is the only Curculionidade species in which the heterochromatin is rich in AT and GC base pairs (Holecová et al. 2013), as several positive marks for DAPI and CMA_3_ were visualized in the majority of its chromosomes.

The analysis of the localization and distribution of rRNA clusters largely contributed toward the cytogenetic characterization of the four *Sitophilus* species analysed. The findings indicate that ribosomal genes are located in a single autosomal pair in three (*S. granarius*, *S. oryzae* and *S. zeamais*) of the four analysed species (different pairs for each species). This corroborates previous reports suggesting that an autosome pair performs as a nucleolus organizer in Coleoptera (Virkki et al. 1984, Colomba et al. 2000, Moura et al. 2003, Gómez-Zurita et al. 2004, Bione et al. 2005, Cabral-de-Mello et al. 2011). This is also the most common pattern observed in the few species of Curculionidae for which the location of the rDNA clusters has been studied, through CMA_3_ staining or silver impregnation (Lachowska 2008, Lachowska et al. 2005, 2006, 2008, 2009, Holecová et al. 2013).

In *S. linearis*, however, positive CMA_3_ and FISH stainings were also detected in the Y chromosome. Data obtained, therefore, evidenced that in this species, the Y chromosome also bears rDNA clusters. To our knowledge, this is the first time that rDNA genes is mapped directly (FISH) on the Y chromosome in Curculionidae, while the presence of rDNA genes on the X or on both sex chromosome (besides autosomes ones) have already been documented in some species of Coleoptera, by FISH analysis (Gómez-Zurita et al. 2004, Bione et al. 2005, Cabrero and Camacho 2008, Cabral de Mello et al. 2011). Furthermore, centromeric, pericentromeric and telomeric clusters were observed in *S. granarius*, *S. oryzae/S*. *zeamais* and in *S. linearis*, respectively. Transposition of genes to new locations, inversions, translocations, ectopic recombination, transposable elements and hybridization without a change in chromosome number are all mechanisms that have already been used to explain this variation in the localization of rDNA genes (Cabrero and Camacho 2008, Panzera et al. 2012, Pita et al. 2013, Golub et al. 2015, Vershinina et al. 2015). Thus, results presented here show that rDNA loci may be considered an important cytogenetic marker for this genus and that cytogenetic analysis on different populations and/or other *Sitophilus* species will certainly contribute to a better understanding of mechanisms responsible for their ribosomal loci variation.

Additionally, CMA_3_ and FISH results revealed fluorescent labels in only one of the homologous of the pair 3 in *S. zeamais*. Although methodological problems cannot be excluded as a source of this variability, it seems unlikely that both techniques would yield the same results, even because they were efficient for the detection of the localization of rDNA genes in the other three *Sitophilus* species. Thus, we believe that this represents a size polymorphism between these homologous and, consequently, that both of them would contain rDNA genes, but that in one of them, the low copy number of ribosomal cistrons (< 10kb [Yiang and Gill 1994]) could not be detected with the probe used here. This suggestion is supported by the fact that this result was found in both populations analysed (Cruzeiro do Sul and Porto Alegre).

### Genome size divergence

The flow cytometry analyses provided a preliminary scenario about the haploid genome size variation among the *Sitophilus* species. The genome size of *S. oryzae* (0.7865 pg) was similar to *S. zeamais* (0.7296 pg), whereas *S. granarius* (0.5533 pg) exhibited a small genome size, and an even smaller was found in *S. linearis* (0.4395 pg). These findings also corroborate the reportedly high intra genus variation in arthropods, as *S. oryzae* has 66% more DNA than *S. linearis*. Although genome size variation is mainly due to variation in the amount of non-coding DNA not necessarily reflecting phylogenetic relationship, this does not seem the case for grain weevils, as we reported here. The variation in DNA content among these four weevil species is consistent and reinforces the phylogenetic relationship among them based on the karyotypes reported here and also on their endosymbionts (Lefevre et al. 2004).

Cytometry data also provided evidence that nuclear DNA content is not proportionally related to either the chromosomal number, or the heterochromatin amount in *Sitophilus* species. In the first case, both smaller genome species (i.e., *S. linearis* and *S. granarius*) exhibit higher chromosome numbers than the species with higher genome sizes (*S. oryzae* and *S. zeamais*). In the second case, *S. linearis* exhibited a similar amount of heterochromatin to both *S. oryzae* and *S. zeamais*, and a larger amount than *S. granarius*, despite the smaller genome size of *S. linearis*. The genome sizes of *Sitophilus* males and females were similar, although three species exhibit the Xyp system, while the tamarind weevil exhibits the neo-XY sex determination system. This findings are suggestive that the genome size variation observed in *Sitophilus* grain weevils may be a result of repetitive DNA sequences (e.g., satellite DNA, transposable elements etc.) accounting for a more complex gene regulation in species with larger genome size, as reported for eukaryotes (Comeron 2006, Biscotti et al. 2015). These larger genome sizes correspond to the more ancestral species, *S. oryzae* and *S. zeamais*, among the grain weevil species. The higher specialization and loss of non-coding DNA may account for the smaller genome size of the more recent grain weevil species, *S. granarius* and *S. linearis*.

The obtained genome size of the *Sitophilus* species were within the previously described range for eight other species of Curculionidae, that include four of the genus *Anthonomus* Germar, 1817 (0.62-0.86 pg – Bárcenas-Ortega 2005, Gregory 2017), one *Dendroctonus* Erichson, 1836 (0.21 pg – Gregory et al. 2013), one *Aramigus* Horn, 1876 (3.32 pg – Normark 1996), one *Lissorhoptrus* LeConte, 1876 (1,00 pg – He et al. 2016) and one *Xyleborus* Eichhoff, 1864 (0.24 pg – Hanrahan and Johnston 2011). The values obtained were also within the constrained value proposed for Gregory (2002) for holometabolous insects (2 pg). However, these values are smaller than that of *Aramigus tessellatus* (Say, 1824) (Normark 1996), a parthenogenetic polyploidy species of Curculionidae with DNA content ranging from 3.32 to 5.02 pg, depending on the analysed lineage (Normark 1996).

Worth noting is also the fact that two genome size estimates were obtained for *S. zeamais* males. Considering that this species may possess from 0-4 B chromosomes, their presence in some individuals explain the difference observed. However, we were unable to carry out cytogenetic and flow cytometry analyses using the same individuals. Consequently, we could neither establish the number of B chromosomes that different individuals possessed nor the contribution of each B chromosome to the whole genome.

### Grain weevil phylogeny

Finally, the parsimony phylogenetic analysis had only mild bootstrap support due to the limited number of informative karyotype characters available, but it does agree with the descriptive analysis of *Sitophilus* karyotype, which provides evidence that *S. zeamais* and *S. oryzae* are phylogenetically closer when compared with *S. granarius* and *S. linearis*. The new finding not previously reported is the higher proximity of *S. granarius* to S. *linearis*, suggesting a common and more recent ancestry for both species. This finding is also consistent with the genome size and the number of chromosomes of these species, the closer association of the granary weevil with stored grains losing its flight ability (Plarre 2010), and with the higher host specialization of the tamarind weevil (Adebayo et al. 2011, Ojo and Omoloye 2015).

The ancient origin (ca. 8.7 million years ago) and closer association between the maize and rice weevils were recently reinforced with comprehensive molecular data (Ojo et al. 2016, Corrêa et al. 2017). This finding is consistent with the ancestral karyotype shared by both species and also resemble that of the granary weevil and their fossil records (Plarre 2010, Corrêa et al. 2017), but is significantly distinct from that of the tamarind weevil. The latter species was recently suggested as clustering with *S. oryzae* and *S. zeamais*, not *S. granarius*, but based only on mtCOI sequence fragment (Devi et al. 2017). Nonetheless, this latter report diverges from the available information on karyotype, genome size, endosymbiont association, and life-history traits of these species (O’Meara 2001, Lefevre et al. 2004, Plarre 2010, and present study). Therefore, the current weight of evidence aided by our findings indicate that the origin of the tamarind weevil is more recent and so is its phylogenetic divergence from the granary weevil and the other stored grain weevils, the maize and rice weevils.

The ancient origin of the grain weevils, likely pre-dating the onset of agriculture in Southeast Asia and the India subcontinent, together with their recent adaptation to stored products, make these earlier invader species useful for tracking grain and trade routes since the Neolithic period between 15,200 and 2,000 BC (Levinson and Levinson 1994, Kenway and Carrott 2006, Smith and Kenward 2011, Panagiotakopulu and Buckland 2017). More abundant fossil information is available for the granary weevil, which is more closely associated with stored commodities due to its inability to fly, but the oldest fossil records are from the maize weevil reinforcing the ancient origin of this species (Levinson and Levinson 1994, Kenway and Carrott 2006, Plarre 2010, Panagiotakopulu and Buckland 2017). Again this is in contrast with the tamarind weevil, whose dispersion is more recent and allegedly associated with the Indian palm (i.e., the tamarind) (Plarre 2010).

## Conclusion

In summary, we were able to describe the karyotype of the tamarind weevil and extend the karyotypic analysis of the maize weevil, allowing a comparative cytogenetic characterization of the four *Sitophilus* grain weevils (*S. granarius, S. linearis, S. oryzae*, and *S. zeamais*). A more complete karyotype-based phylogenetic analysis of these four species, aided by the quantification of genome size in each, shed light on the conflicting phylogeny of the grain weevil species. The ancestral and closer phylogenetic association between *S. zeamais* and *S. oryzae* was recognized, as was the more recent cluster encompassing *S. granarius* and *S. linearis* and a shared ancestral relationship.
